# The Broad Host Range Plant Pathogen *Dickeya dianthicola* Shows a High Genetic Diversity

**DOI:** 10.3390/microorganisms10051024

**Published:** 2022-05-13

**Authors:** Jacques Pédron, Jan M. van der Wolf, Perrine Portier, Emma Caullireau, Frédérique Van Gijsegem

**Affiliations:** 1 Institute of Ecology and Environmental Sciences-Paris, Sorbonne Université, INRAE, 4 Place Jussieu, F-75252 Paris, France; jacques.pedron@upmc.fr; 2 Wageningen University & Research, P.O. Box 16, 6700 AA Wageningen, The Netherlands; jan.vanderwolf@wur.nl; 3 University Angers, Institut Agro, INRAE, IRHS, SFR QUASAV, CIRM-CFBP, F-49000 Angers, France; perrine.portier@inrae.fr (P.P.); emma.caullireau@gmail.com (E.C.)

**Keywords:** soft rot *Pectobacteriaceae*, plant pathogen, comparative genomics, potato, ornamentals

## Abstract

The wide host range phytopathogen *D. dianthicola*, first described in ornamentals in the 1950s, rapidly became a threat for potato production in Europe and, more recently, worldwide. Previous genomic analyses, mainly of strains isolated from potato, revealed little sequence diversity. To further analyse *D. dianthicola* genomic diversity, we used a larger genome panel of 41 isolates encompassing more strains isolated from potato over a wide time scale and more strains isolated from other hosts. The phylogenetic and pan-genomic trees revealed a large cluster of highly related genomes but also the divergence of two more distant strains, IPO 256 and 67.19, isolated from potato and impatiens, respectively, and the clustering of the three strains isolated from Kalanchoe with one more distinct potato strain. An SNP-based minimal spanning tree highlighted both diverse clusters of (nearly) clonal strains and several strains scattered in the MST, irrespective of country or date of isolation, that differ by several thousand SNPs. This study reveals a higher diversity in *D. dianthicola* than previously described. It indicates the clonal spread of this pathogen over long distances, as suspected from worldwide seed trading, and possible multiple introductions of *D. dianthicola* from alternative sources of contaminations.

## 1. Introduction

The worldwide spread of plant pathogens is mainly due to the trade of plants for planting as well as jumps of pathogens between plant species. This phenomenon has been observed for decades for species of the genera *Pectobacterium* and *Dickeya* [1]. These genera, belonging to the *Pectobacteriaceae*, cause soft rot, wilts, stunting and cankers on several crops, vegetables, ornamental plants and trees [1]). Their most important virulence factor is the production and secretion of a battery of plant cell wall degrading enzymes that provoke the maceration of plant tissues, leading to cell lysis and release of the cell content. However, virulence also relies on several other factors that allow these bacteria to adapt to environmental changes encountered in planta and to face the stresses produced by plant defence responses [2].

*D. dianthicola* is one of the broad host range species within the group of soft rot *Pectobacteriaceae* (SRP) [3]. It was first described in a carnation (*Dianthus*) outbreak in the UK, the Netherlands and Denmark [4], and thereafter in several other European countries [5,6,7,8]. Since then, *D.* *dianthicola* has been reported worldwide [3]. It affects a dozen other crops, mainly ornamentals, but also chicory, tomato and potato [1,9].

Although reports of *D. dianthicola* (at the time grouped with all *Dickeya* under the name *Erwinia chrysanthemi*) on potato in Europe date back to the 1970s, losses caused by this pathogen have been generally low and sporadic in most countries [10]. An exception is Switzerland and the Netherlands, where *D. dianthicola* has been one of the dominant agents of blackleg and soft rot in potato since the 1980s until 2000, after which *D. solani* became prevalent; since 2014, *P. brasiliense* dominates [3,11,12]. In the past decade, potato blackleg caused by *D. dianthicola* has been reported in several locations worldwide. It was found in Pakistan [13] and Morocco [14]. In 2016, it caused a severe outbreak in the US [15,16] and, in 2017, in Western Australia [17].

MLSA analyses based on housekeeping gene sequences showed that little sequence diversity was found in *D. dianthicola* strains [8,12,16,18,19,20]. This high relatedness was confirmed by whole-genome analyses [19,20,21]. In particular, Ge et al. (2021) [21] showed that *D. dianthicola* strains recently isolated from potato in the US were mostly clonal, indicating a single introduction as the main cause of the recent US outbreak. However, there is a bias in these studies due to the analysis mainly of strains isolated from only one host, potato, and only from Europe and the US. There is indeed often a strong association between genetic diversity and the host of the pathogen. This is illustrated by strain 67.19 (recently isolated from impatiens in the US), which is more diverse, only sharing 97% average nucleotide identity (ANI) with other *D. dianthicola* genomes [22], pointing to a possible higher diversity in strains isolated from different plant hosts.

In this context, the goal of this study is to analyse *D. dianthicola* genomic diversity using a larger strain panel encompassing more strains isolated from potato over a wide time scale and more strains isolated from hosts other than potato. We also address the questions of the relationship between genetic variation and possible host specialisation and of the evolutionary history of this species.

## 2. Materials and Methods

### 2.1. dnaX-leuS-recA Phylogeny of Dickeya Strains from CIRM-CFBP

For the 32 strains of *D. dianthicola* listed in Table S1, portions of the genes *recA*, *leuS* and *dnaX* were sequenced, following the protocol described for *Pectobacterium* [23]. Sequencing of PCR products was performed by Genoscreen (Lille, France). The consensus sequences for each gene for each strain were extracted from forward and reverse sequences assembly using Geneious Pro v. 9.1.8 (http://www.geneious.com/) (accessed on 27 April 2022). The sequences were then aligned and trimmed using BioEdit v. 5.0.6. A phylogenetic tree was constructed with concatenated alignments of all genes with MEGA 7.0.26 [24] using the Neighbour-Joining method with 1000 bootstrap replicates, and the evolutionary distances were computed using the Kimura 2-parameter method.

### 2.2. DNA Extraction, Genome Sequencing and Assembly

The bacterial genomes used in this study are presented in Table 1. Total bacterial DNA was extracted from pure bacterial cultures using the Wizard genomic DNA purification kit (Promega), following the manufacturer’s protocol.

DNA samples of IPO strains were sequenced using short-read sequencing (Illumina, San Diego, CA, USA). Random sheared shotgun library preparation was performed using the Truseq Nano DNA Library Prep kit (dual indexing) following the manufacturer’s protocol. The samples were loaded on a paired-end flowcell using the Hiseq PE cluster kit V4 (Illumina). One hundred twenty-five bp paired-end sequences were generated on a Hiseq 2500 (Illumina, San Diego, CA, USA).

Genome sequencing of CH and CFBP strains was performed at the next-generation sequencing core facilities of the Institute for Integrative Biology of the Cell (Avenue de la Terrasse 91190 Gif-sur-Yvette France). Nextera DNA libraries were prepared from 50 ng of high-quality genomic DNA. Paired-end 2 × 75 bp sequencing was performed on an Illumina NextSeq500 apparatus with a High Output 150 cycle kit. CLC Genomics Workbench (Version 9.5.2, Qiagen Bioinformatics) was used to assemble reads. Final sequencing coverage was between 49 and 79. Coding sequences were predicted using the RAST server [25] with the Glimmer 3 prediction tool [26].

### 2.3. Genome Analysis

Pairwise comparison of the genomes was computed using the average nucleotide identity (ANI) Pyani python module (https://github.com/widdowquinn/pyani (accessed on 27 April 2022) [27] with the blast algorithm (ANIb). The species threshold was set at 96%.

Orthologous sequences were clustered into homologous families using the SiLix software package [28], with a 70% identity threshold and at least 80% overlap. Strain-specific and clade-specific gene families and gene families absent in only one of the analysed genomes were extracted from the SiLix output. For the construction of MLSA trees, common genes (defined as genes present in all strains meeting the criteria of 70% identity threshold and at least 80% overlap) were aligned using MUSCLE [29] software and were filtered using the GBLOCK tool [30]. The alignments were used for building a phylogenetic tree with the BioNJ algorithm with SeaView software [31], with 200 bootstrap replications.

Pan-genome clustering: A hierarchical clustering was performed for the pan-genome, as described by Meric et al. [32]. Briefly, a presence/absence matrix for all genes (pangenome) was constructed; Manhattan distances were calculated and used for hierarchical clustering to generate the tree. Thus, unlike MLSA trees that only take into account variations in the core-genome, pan-genome clustering is based on the similarities (presence or absence) of the totality of the gene repertoires. Protein families only occurring in a single genome (singletons) were not included in the analysis. The Mega7 tool [24] was used to visualise the phylogenetic tree with the BioNJ algorithm.

Rarefaction (core genome) and accumulation (pan-genome) curves were calculated with R scripts as described in [32].

### 2.4. Minimum Spanning Tree Analysis

SNPs were extracted from the aligned coding sequences of the core genome. For representing the possible evolutionary relationships between strains (minimum spanning tree), we used the online version of the software PHYLOViZ [33].

## 3. Results

### 3.1. Panel of Genomes Analysed in This Study

This study is based on the analysis of 41 *D. dianthicola* genomes (Table 1).

**Figure 1:**
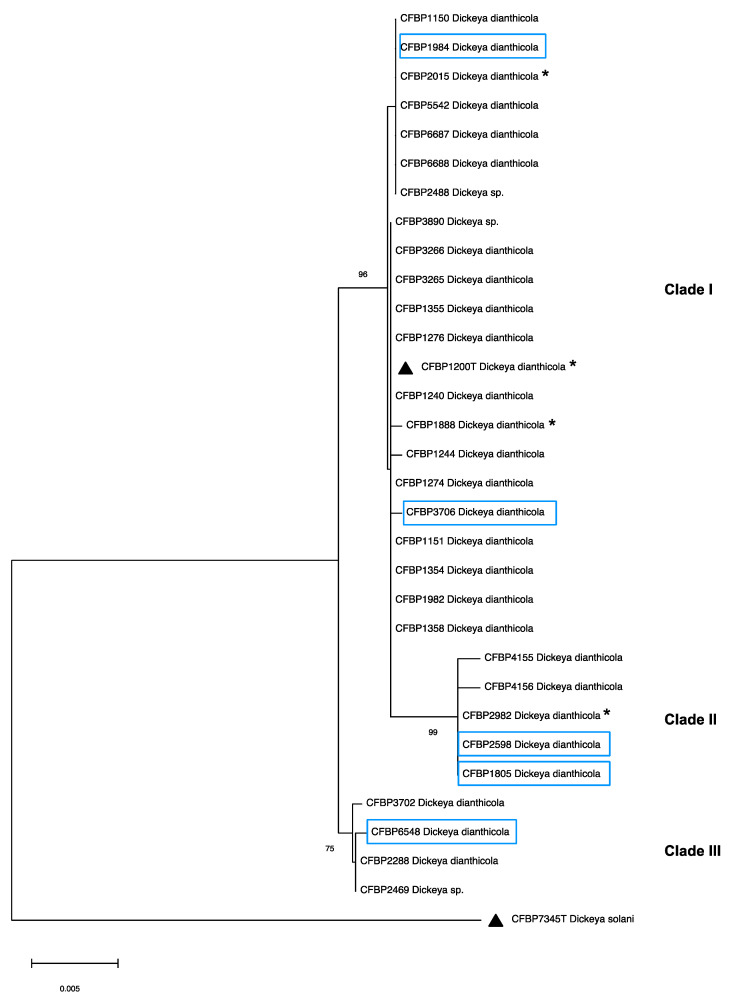
Phylogenetic tree of *Dickeya dianthicola* strains from CIRM-CFBP constructed from partial sequences from *dnaX*, *leuS*, and *recA* housekeeping genes. Strain *D. solani* CFBP 7345 was used as the outgroup. Bootstraps values are shown when superior to 70. The black triangles indicate the type strains. The strains identified by a star are strains for which a complete genome sequence already exists in the public databases. The strains in a square were chosen for complete genome sequencing in this study.

In total, 17 of them were retrieved from the NCBI Resource Centre; they consist of 14 strains isolated from potato in 7 different countries on 4 continents and 3 strains isolated from ornamentals, including the type strain NCPPB 453 (CFBP1200). Fifteen genomes were sequenced from strains originating from the Wageningen University & Research bacteria strain collection (IPO strains) and four genomes from the collection of the Swiss Genoscope Institute (CH strains). The Swiss strains were selected from a wider panel of *D. dianthicola* strains present in the CH collection among the ones harbouring some variations in their *gapA* sequences, widely used to classify *Dickeya* and *Pectobacterium* strains [20,34]. Seventeen strains have been isolated from potato, whereas IPO 1003 originated from Belgian chicory and IPO3797 from Sedum. As most of these 36 strains originated from potato, the panel was extended with strains of other plant hosts present in the French Collection for Plant-associated Bacteria, CIRM-CFBP. For the selection of the CFBP strains, an MLSA analysis was performed on the basis of *recA*, *leuS* and *dnaX* housekeeping genes. The MLSA tree showed that the *D. dianthicola* strains of the CIRM-CFBP collection are split into three clades (Figure 1).

The first clade comprises a relatively homogenous group of strains isolated from various plants and a subclade (Clade II) that groups all strains isolated from Kalanchoe. The third clade groups four strains: one isolated from chicory, one from *cynara* and two from potato. For whole-genome sequencing, we selected two strains of clade I isolated from chicory (CFBP 3706) or carnation (CFBP 1984), two strains of clade II isolated from Kalanchoe (CFBP 1805 and CFBP 2598), and one strain from the third clade isolated from chicory (CFBP 6548) (Figure 1).

The type strain NCPPB453 was isolated in 1956, while the strains of our panel have been sampled from the 1970s up to 2019; thus, our panel comprised strains isolated over seven decades, originating from five continents.

### 3.2. D. dianthicola Diversity

Calculation of average nucleotide identity (ANI) values with the type strain NCPPB 453 confirmed the assignation to the species *D. dianthicola* for all analysed strains (Table 1 and Table S2). Most strains shared ANI values higher than 99%, both with the type strain and, between them, showing a high relatedness. The most distant strains IPO 256 isolated from potato in The Netherlands and 67-19 isolated from impatiens in the US share ANI values from 97.0% to 98.4% with all other *D. dianthicola* genomes.

**Figure 2:**
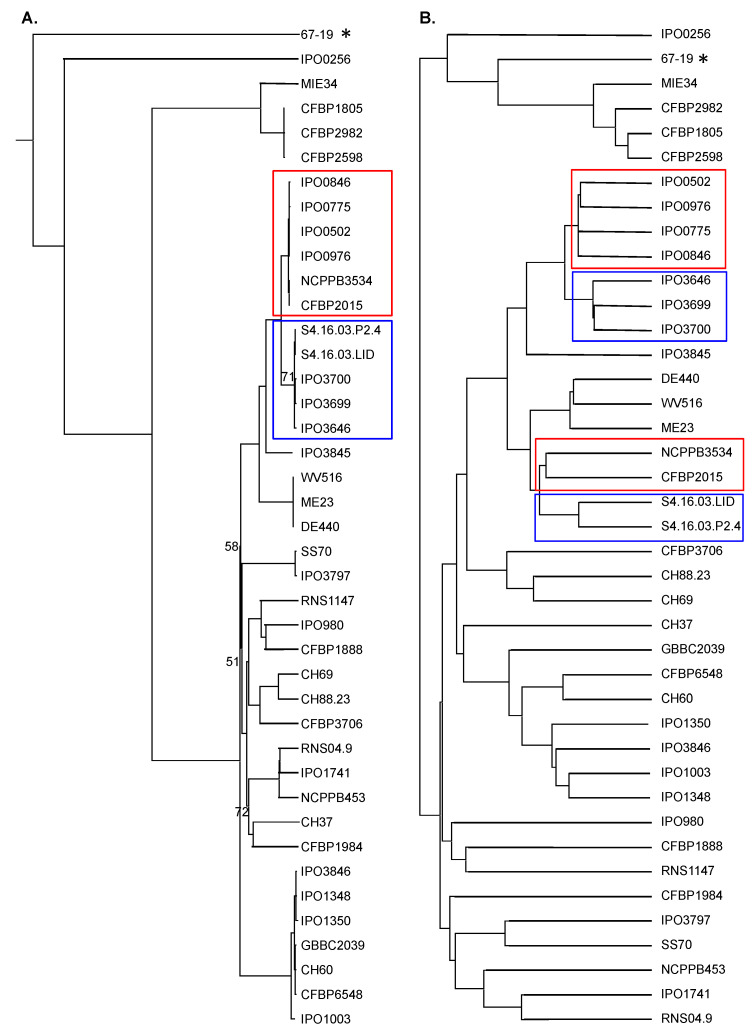
Phylogeny of *Dickeya dianthicola*. (**A**). Phylogenic tree built up from the concatenated sequences of 2996 homologous protein sequences (19,293 variable sites). One hundred bootstrap replicates were performed to assess the statistical support of each node. Only bootstraps values below 100% are presented. (**B**). Pangenome tree: distance was calculated from a presence/absence matrix of the pangenome (see Section 2). Stars and rectangles indicate differences between both trees.

A whole-genome MLSA phylogenomic tree built up from concatenated sequences of 2996 core proteins (Figure 2A) confirmed the high relatedness of most strains of our panel and the divergence of the two more distant strains IPO 256 and 67.19. It also highlighted the clustering in a separate clade of four strains (the three strains isolated from Kalanchoe CFBP 2982, CFBP 1805 and CFBP 2598 and the potato strain MIE34). These four strains are closely related (99.6–99.9% ANI) and more distant from other strains (98.4–98.7% ANI). The chicory strain CFBP6548, a member of class III in the MLSA tree (Figure 1), is grouped with several potato strains. As already indicated by ANI values (99.2–99.4%), this cluster is closely linked to members of the main clade I (Figure 2A). 

In order to further address the relationships between the different *D. dianthicola* strains, we compared the protein-coding sequences of the 41 genomes using the SiLix gene family clustering tool. Proteins were classified as homologous to others in a given family if the amino acid identities were above 70%, with 80% minimal overlap.

**Figure 3:**
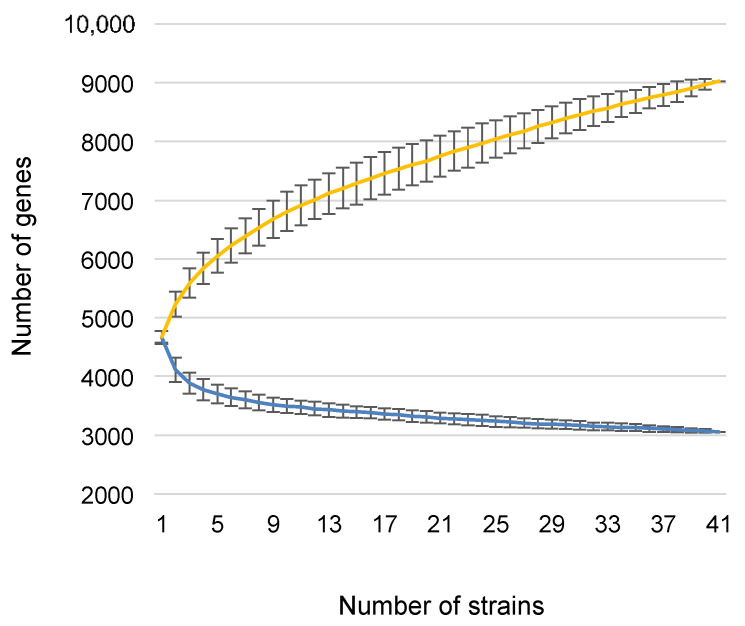
Rarefaction and accumulation curve estimates of *Dickeya dianthicola* core and pan-genomes. The number of shared genes (blue line) and the total number of genes (yellow line) were determined as genome sampling increased. Comparisons were made based on matrices of gene presence/absence. Randomised genome sampling was carried out 100 times to obtain the average number of genes for each sample comparison number and standard deviations.

All *D. dianthicola* strains share 3055 protein families representing 63% to 69% of the genome content. In particular, they share most of the arsenal of virulence genes characterised in the model strain *D. dadantii* 3937. They all possess the genes involved in pectin degradation except that they harbour a truncated form of the pectate lysase PelA encoding gene and do not possess the gene encoding the predicted polygalacturonase PehK. They are all lacking the gene encoding the protease PrtG, one of the four proteases secreted by the type I Prt protein secretion system. They possess only one of the two avirulence-related *avrL*-*avrM* genes present in the model strain *D. dadantii* 3937. Genes involved in resistance to the various stresses that might be encountered during plant infection are also present in all *D. dianthicola* strains, including those involved in oxidative stress resistance, such as the *kat* and *sod* genes encoding catalases and superoxide dismutases, in resistance to antimicrobial peptides (*arnB-T*, *sapA-E*) and in siderophore synthesis and uptake (*acsF-A* and *cbrABCDE* for achromobactin and *fct-cbsCEBA* for chrysobactin). The rarefaction curve presented in Figure 3 shows that the *D. dianthicola* core genome reaches a low level plateau with the deletion of only about ten protein families per additional genome, indicating the *D. dianthicola* core genome is almost closed.

The pan-genome that comprises all protein families present in *D. dianthicola* members includes 9025 protein families for the 41 genomes studied. It is clearly still expanding, being far from reaching a plateau (Figure 3). However, the SiLix analysis revealed that most strains only harbour, at most, a few dozen specific genes (Table 1). This depicts the fact that several strains belong to clusters grouping highly related genomes, with only rare genes present in only one strain. The two external strains 67.19 and IPO256, on their part, carry 502 and 240 strain-specific genes, respectively.

### 3.3. Relatedness of D. dianthicola Strains by SNP Analysis

**Figure 4:**
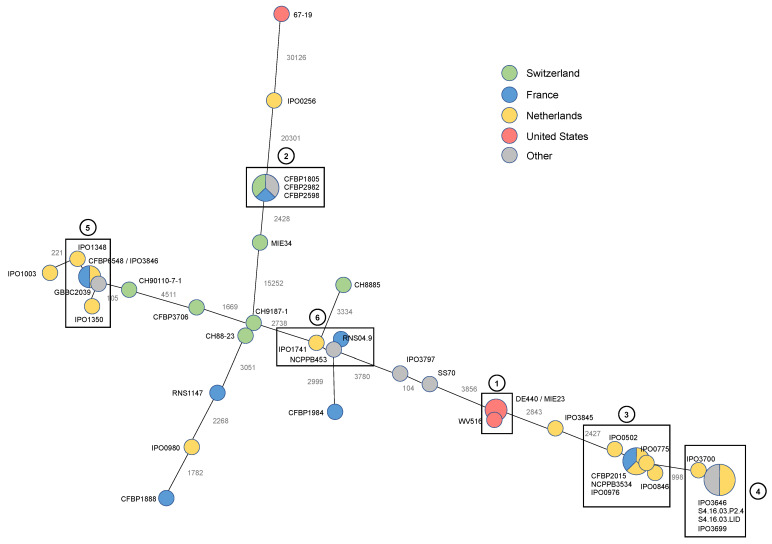
Minimum spanning tree based upon whole-genome SNP analysis. The tree is based upon 53,292 SNPs. The length of each branch (log scale) expressed in SNP numbers is indicated. The squares and numbers indicate the clusters of (nearly) clonal strains.

To tackle *D. dianthicola* evolution and epidemiology, we constructed a Minimum Spanning Tree (MST) based on the whole set of single nucleotide polymorphisms (SNPs) present in the core genome identified in our strain panel (Figure 4).

This MST revealed several clusters of strains that do not harbour any polymorphism or only differ by a few SNPs. Two of these clusters could be clearly linked to defined outbreaks: cluster 1 grouping the potato strains isolated during the recent US outbreak [22,35] and cluster 2 grouping the strains isolated from Kalanchoe in Europe during the late 1970s-early 1980s outbreak. Evidence of clonal spread is also notable in cluster 3, which includes potato strains from the Netherlands and France isolated from 1975 to 1991. Cluster 4 groups more recent strains (isolated from 2013) that appeared to emerge from this Cluster 3. Cluster 5 includes a bit more loosely connected strains isolated from the Netherlands, France, Belgium and Switzerland in potato or Belgian chicory from 1988 to 2009. More intriguing is Cluster 6, which groups the type strain NCPPB353 isolated in 1956 from carnation with two almost clonal potato strains isolated either in the Netherlands in 1992 (IPO1741) or in France in 2004 (RNS04.9).

Other strains are scattered in the MST, irrespective of country or date of isolation, pointing to multiple introductions of *D. dianthicola* in Europe. This is exemplified by the three Swiss potato strains CH88-23, CH9187-1 and CH8885. Indeed, the two first ones, isolated in 1988 and 1991, respectively, only harboured three SNPs, indicating a filiation, whereas CH8885, isolated in the same year as CH88-23, was not connected (Figure 4).

### 3.4. Diversity in D. dianthicola Accessory Genome

The whole-genome MLSA tree construction is based on genes of the core genome; it does not analyse the relatedness of the variable part of the genome, the accessory genome. To analyse this accessory genome, we performed a pan-genome clustering analysis that builds a hierarchical clustering tree based on the proportion of the presence/absence status of each gene family in each genome (Figure 2B). The main difference between the two trees is the position of the external strain 67.19. In the pan-genome tree, it groups with the cluster of strains isolated from Kalanchoe. For the other strains, there are only a few differences between both trees. In the phylogenetic tree (Figure 2A), the subclade highlighted in red groups early potato strains isolated from 1975 to 1991, while the subclade highlighted in blue groups more recent strains isolated from 2009 to 2016. This clustering, following the year of isolation, is not conserved in the pan-genome tree: these two subclades, present in the phylogenetic tree, are split and rearranged into two other subclades, one grouping seven IPO strains belonging to either the “red” or “blue” subclades, while the second one groups three IPO strains and two strains isolated in Morocco, belonging respectively to the phylogenetic “red” and “blue” subclades, with the cluster of strains isolated in the US (Figure 2B).

### 3.5. Are There Genes Related to Host Specificity in D. dianthicola?

Besides many strains isolated from potato, our strain panel includes two sets of strains isolated either from the ornamental Kalanchoe or from Belgian chicory. The three Kalanchoe strains are highly related and cluster in distinct tree branches, both in their core genomes (100% ANI values between them, Figure 2A and Figure 4) and in their accessory genomes (Figure 2B). This prompted us to analyse if these three strains share genes that might be involved in host specificity. Only the 16 genes present in these 3 strains and absent in all other *D. dianthicola* genomes were analysed. Eleven of these genes encode hypothetical proteins, and two are related to mobile genetic elements. In comparison, the potato strain MIE34 and the Impatiens strain 67.19, which belong to the same pan-genomic tree cluster (Figure 2B), shared with the Kalanchoe strains 66 homologous protein families that are absent in all other *D. dianthicola* genomes (Table S3). Besides 33 and 6 genes encoding, respectively, hypothetical proteins and proteins related to mobile genetic elements, protein families specific to this cluster include 8 regulators and 5 proteins involved in transport. Only eight protein families shared by the strains of this cluster, which are isolated from ornamentals (Kalanchoe and impatiens), are absent from the potato strain MIE34. This points more to differences related to phylogeny rather than host specificity.

The three strains isolated from chicory are more distant from each other, both in their core and accessory genomes (Figure 2). They do not share any specific protein families that are absent in all other *D. dianthicola* genomes.

## 4. Discussion

Previous studies of *D. dianthicola* genomic diversity [12,19,21] focused on strains isolated from potato. They highlighted the high relatedness of the strains that shared ANI values higher than 99% and even the clonality of most strains isolated during the outbreak that have ravaged the US since 2015. These analyses suffered, however, from a bias due to sampling generally only from potato.

In this paper, we extended the analysis of *D. dianthicola* species diversity by including the recently described genomes of 2 strains isolated from ornamentals (CFBP 2982 and 67.19) as well as new genomes of 17 strains isolated from potato in the Netherlands and in Switzerland and 7 strains isolated from other hosts. This genome panel revealed wider *D. dianthicola* diversity. Indeed, besides the low ANI values of 97% recently reported between *D. dianthicola* strains isolated from potato and strain 67.19 isolated from Impatiens [22], strain IPO0256 isolated from potato is also more divergent (ANI values around 98.5%), with a clade grouping the three strains isolated from Kalanchoe and the potato strain MIE34 (ANI values slightly lower than 99%). We thus observed an extension of diversity related to host range when analysing strains isolated from other plant hosts. This is, however, not a general rule since strains isolated from chicory are closely related to potato isolates and, conversely, the second more distant strain, IPO256, was isolated from potato.

Besides whole-genome-based MLSA, we analysed whole core genome SNPs to define the genetic diversity extent in *D. dianthicola* that may provide insights into the evolution, transmission and molecular epidemiology of this pathogen. Our SNP-based phylogenetic analysis using the whole set of SNPs is congruent with the clusters defined by whole-genome-based MLSA. It revealed interesting features of the possible routes of introduction of *D. dianthicola* in different countries with a mix of clonal spread and multiple introductions of the pathogen (Figure 4). Only for the blackleg outbreak in the US in 2015 a clonal structure of the *D. dianthicola* populations was found [15,21]. Notably, the MST analysis placed the two more diverse strains, 67.19 and IPO256, close to the cluster of the Kalanchoe strains (Figure 4), and such proximity of strain 67.19 and Kalanchoe strains in their accessory genomes has also been revealed in the pan-genome tree. We have, however, to keep in mind that MST analysis shows the proximity of strains and is not directly linked to evolution, especially in this case where the numbers of SNPs between the genomes are very high. Furthermore, Kalanchoe and Impatiens, the 67.19 hosts, are both ornamentals, but Kalanchoe belongs to the Saxifragales while Impatiens belongs to the Asterids, like the Solanale, including potato, making minor a possible role of host characteristics in this proximity. Clearly, more strains isolated from these two hosts should be analysed to apprehend possible clues on this apparent proximity. 

Interestingly, *D. dianthicola* strains that have been isolated for as long as a decade apart from different countries (see, for example, the CFBP2015 strain isolated in France in 1975 versus the NCPPB3534 strain isolated in The Netherlands in 1987) are almost clonal, differing by only 15 SNPs (Figure 4). This highlights the high genomic stability for some members of this species. For *P. brasiliense,* Jonkheer et al. (2021) reported such a high relatedness of several strains within a clade of strains isolated from different locations, besides a clade of more diverse strains [36]. In this organism, clonality was related to high aggressiveness. It would be interesting to analyse if such a correlation is also present in *D. dianthicola,* pointing to a possible role of a high ecological fitness in the persistence of this clonal population. Clonal spreads are also observed for the *D. dianthicola* outbreak that impaired Kakanchoe production in Europe in the 1970s–1980s as well as for different potato blackleg outbreaks in various geographical areas, such as found for Cluster 4, with a spread even up to Morocco (Figure 4). This points to the important role of long-distance seed trading dissemination in the propagation of this pathogen. However, several other *D. dianthicola* strains are only loosely connected even if isolated in the same country and/or the same year, pointing to distinct sources of contamination. It was for a long time proposed that introductions of new variants in potato may arise from a jump of *Dickeya* species from ornamentals [9,18]). This hypothesis is supported by the high relatedness of the type strain NCPPB453 from Dianthus, but also the loosen proximity of the Kalanchoe isolates or the Sedum isolate IPO3797 with various strains isolated in potato. In a recent report, Aono et al. [37] concluded that potato blackleg infection in the field may be caused by the transmission of *D. dianthicola* from infested Asteraceae weeds to potato plants through surface water flow, highlighting another propagation route arising from the environment.

Previous reports describing some host specificities for *D. dianthicola* [38] prompted us to analyse if our genome analysis might highlight genes that might be involved in host specificity. In this study, no host specificity studies were undertaken, and we can only use the information on the host from which the strain was isolated as a lead. No indications for genes involved in host specificity were identified in *D. dianthicola* strains isolated from Belgian chicory that are more close to some potato isolates than potato strains to each other. This points more to cross-contaminations that may occur in the field since chicory can be part of crop rotations with potato. Kalanchoe *D. dianthicola* isolates shared a dozen proteins, most being hypothetical, that are not present in other *D. dianthicola* strains. Since the analysed Kalanchoe isolates are clonal, more strains isolated from this host should be analysed to assess if such genes might be involved in host specificity or just reflect the genetic relatedness between these strains.

In conclusion, our study, including the genomic comparison of *D. dianthicola* strains isolated from potato and from other hosts, has revealed a higher diversity in this species than previously described. Minimum spanning tree analysis highlighted the clonal spread of the pathogen over long distances, while multiple introductions of the pathogen, even in the same country, are also likely. To have insight into the putative genes involved in host specificity, more strains isolated from Kalanchoe, preferably from outside of Europe, need to be analysed. Analysis of isolates from South America (the origin of potato) or from current outbreaks in Asia and Oceania could also reveal an even higher diversity of this pathogen.

## Figures and Tables

**Table 1:** Genomes of *D. dianthicola* analysed in this study.

Genomes	Host	Country of Isolation	Year of Isolation	Source	# Contigs	# CDS	# Specific Genes *	ANI Values ** (%)
WV516	*Solanum tuberosum*	US	2016	NCBI	103	4795	42	99.4
SS70	*Solanum tuberosum*	Pakistan	2017	NCBI	62	4665	24	99.5
NCPPB3534	*Solanum tuberosum*	The Netherlands	1987	NCBI	41	4663	38	99.4
ME23	*Solanum tuberosum*	US	-	NCBI	complete	4790	3	99.4
IPO_980	*Solanum tuberosum*	The Netherlands	1991	NCBI	52	4493	57	99.5
GBBC2039	*Solanum tuberosum*	Belgium	-	NCBI	1	4607	200	99.4
DE440	*Solanum tuberosum*	US	2016	NCBI	55	4792	28	99.4
RNS1147	*Solanum tuberosum*	France	2011	NCBI	78	4904	85	99.5
RNS04.9	*Solanum tuberosum*	France	2004	NCBI	complete	4567	12	1.00
CFBP2015	*Solanum tuberosum*	France	1975	NCBI	55	4666	2	99.4
MIE34	*Solanum tuberosum*	Switzerland	2013	NCBI	94	4568	23	98.8
S4.16.03.P2.4	*Solanum tuberosum*	Morocco	2016	NCBI	101	4768	8	99.4
S4.16.03.lid	*Solanum tuberosum*	Morocco	2016	NCBI	108	4775	8	99.4
CFBP1888	*Solanum tuberosum*	France	1978	NCBI	67	4752	47	99.5
NCPPB_453	*Dianthus*	UK	1956	NCBI	1	4477	46	-
CFBP2982	*Kalanchoe*	France	1978	NCBI	90		17	98.7
67.19	New *Guinea Impatiens*	US	2019	NCBI	1	4637	502	97.3
IPO0256	*Solanum tuberosum*	The Netherlands	1975	This work	212	4696	240	98.5
IPO0502	*Solanum tuberosum*	The Netherlands	1979	This work	161	4726	4	99.4
IPO0775	*Solanum tuberosum*	The Netherlands	1984	This work	188	4702	12	99.4
IPO0846	*Solanum tuberosum*	The Netherlands	1987	This work	224	4718	35	99.4
IPO0976	*Solanum tuberosum*	The Netherlands	1991	This work	194	4711	15	99.4
IPO1003	*Cichorium intybus*	The Netherlands	1988	This work	214	4806	10	99.4
IPO1348	*Solanum tuberosum*	The Netherlands	1993	This work	209	4784	12	99.4
IPO1350	*Solanum tuberosum*	The Netherlands	1994	This work	555	4876	216	99.4
IPO1741	*Solanum tuberosum*	The Netherlands	1992	This work	148	4508	11	1.00
IPO3646	*Solanum tuberosum*	The Netherlands	2013	This work	176	4814	7	99.4
IPO3699	*Solanum tuberosum*	The Netherlands	2013	This work	181	4817	3	99.4
IPO3700	*Solanum tuberosum*	The Netherlands	2013	This work	184	4826	10	99.4
IPO3797	*Sedum*	The Netherlands	2010	This work	120	4645	26	99.5
IPO3845	*Solanum tuberosum*	The Netherlands	2013	This work	101	4632	25	99.4
IPO3846	*Solanum tuberosum*	The Netherlands	2009	This work	109	4777	4	99.4
CH88.23	*Solanum tuberosum*	Switzerland	1988	This work	87	4898	25	99.5
CH8885	*Solanum tuberosum*	Switzerland	1988	This work	113	4746	68	99.5
CH90110-7-1	*Solanum tuberosum*	Switzerland	1990	This work	119	4798	11	99.4
CH9187-1	*Solanum tuberosum*	Switzerland	1991	This work	126	4896	37	99.5
CFBP1805	*Kalanchoe*	Denmark	1977	This work	200	4706	48	98.6
CFBP1984	*Dianthus*	France	1972	This work	126	4611	66	99.5
CFBP2598	*Kalanchoe*	Switzerland	1982	This work	168	4723	33	98.6
CFBP3706	*Cichorium intybus*	Switzerland	1986	This work	148	4713	46	99.6
CFBP6548	*Cichorium intybus*	France	1994	This work	158	4857	91	99.4

* Specific genes are genes present only in the given strain; ** ANI values with the type strain NCPPB453.

## Data Availability

Publically available genomes were retrieved from the microbial genomes section at NCBI. The genomes produced in this study have been deposited at NCBI under accession numbers JALDNP000000000 to JALDOM000000000.

## Supplementary Materials

The following supporting information can be downloaded at: https://www.mdpi.com/article/10.3390/microorganisms10051024/s1, Table S1: *D. dianthicola* strains from CIRM-CFBP; Table S2: ANI values for genomes analysed in this study; Table S3: Protein families specific to strains of the Kalanchoe cluster. Strains from CIRM-CFBP can be accessed at https://cirm-cfbp.fr.
